# Supplementary material for: Research on reachable set boundary of neutral system with various types of disturbances
Source: PLoS One. 2025 Jan 16;20(1):e0317398. doi: 10.1371/journal.pone.0317398 (PMC11737737; doi:10.1371/journal.pone.0317398)
Supplement: S1 File — (PDF) [file pone.0317398.s001.pdf]

%%%% The main procedures in this paper can be referred to as follows

%% According to the linear matrix inequality given in Theorem 1, we can find the appropriate P1 matrix through LMI toolbox in Matlab. The specific steps are as follows:：

```
A = [-2 0; 0 -0.9];

B = [-1 0; -1 -1];

C = [0 0; 0 0];

D = [-0.5; 1];

tau = 0.1;

h = 0.7;

w=0.2*sin(t);



P1 = sdpvar(2,2);

P2 = sdpvar(2,2);

P3 = sdpvar(2,2);

Q11 = sdpvar(1,1);

Q12 = sdpvar(1,2);

Q22 = sdpvar(2,2);

X = sdpvar(2,2);

Y = sdpvar(2,2);

Z = sdpvar(2,2);

R = sdpvar(1,1);

G = sdpvar(2,2);

S1 = sdpvar(1,1);

S2 = sdpvar(1,1);

Md = sdpvar(2,2);
```

```
Phi33 = h*Q22-Q12-Q12'-min((1-h)*exp(-alpha*h), 1-h)*Md-exp(-alpha*h)*S1;

LMI1 = [alpha*P1+alpha*X+P2*A+A'*P2+Md-exp(-alpha*h)*S1 P1-P2'+A'*P3+X+Y
P2'*B+Q12+exp(-alpha*h)*S1 P2'*C-Y zeros(2,1) alpha*Y P2'*D;
        P1-P2+A*P3'+X+Y' Q11*(-exp(-alpha*tau)) R*eye(2) Q12' zeros(2,1) Y'+Z P3'*B;
        B*P2+Q12*eye(2) Phi33 zeros(2,1) zeros(2,1) zeros(2,1) zeros(2,1);
        P3*B' Q12 zeros(1,1) -exp(-alpha*tau)*R zeros(1,2) -Z zeros(1,1);
        zeros(1,5) -exp(-alpha*h)*S2 zeros(1,2);
        alpha*Y zeros(1,5) alpha*Z-exp(-alpha*tau)*G zeros(1,1);
        D'*P2 zeros(1,5) zeros(1,2) (-alpha/w^2)*eye(1)];

LMI2 = [X Y; Y' Z+1/tau*exp(-alpha*tau)*R]>=0;
LMI3 = [Q11 Q12; Q12' Q22]>=0;
LMI4 = [P1]>0;

sol = optimize([LMI1<=0, LMI2, LMI3, LMI4], [], sdpsettings('solver','sedumi'));

P1 = value(P1)

P=[0.6616 0.2587; 0.2587 2.1984]

% Characteristic decomposition of p
[V, D] = eig(P);

% Calculate the semi-axis length of ellipsoid
a = sqrt(D(1, 1));
b = sqrt(D(2, 2));
```

```
% Calculate the rotation angle theta
theta = atan2(V(2, 1), V(1, 1));

% Parametric equation for generating ellipse
t = linspace(0, 2 * pi, 100);
x = a * cos(t);
y = b * sin(t);
% Define the value range of state variables.
x1_range = [-2, 2];
x2_range = [-2, 2];
% Application rotation
x_rot = x * cos(theta) - y * sin(theta);
y_rot = x * sin(theta) + y * cos(theta);
% Define step size
dx = 0.1;

% Generate state grid
[x1_grid, x2_grid] = meshgrid(x1_range(1):dx:x1_range(2), x2_range(1):dx:x2_range(2));

% Calculate the 1-norm distance of each point in the state grid
d = abs(x1_grid) + abs(x2_grid);

% Mark the point whose 1 norm distance is less than or equal to 1 as the reachable state.
reachable = d <=1;

clc;
clear;
```

```
%%%%%%%%%%%%%%% Parameter setting

A =[                0, 0,    -32.0,        -78.12,      0;

                    0, 0, 7850.0,                 0,      0;

   -1.5183e-10, 0,            0,    0.067381,      0;

     1.5183e-10, 0,            0, -0.067381, 1.0;

                    0, 0,            0,          3.381,      0];


B =[           28.878,        0;

                    0,             0;

     0.00023761,             0;

   -0.00023761,             0;

          0.14515, -3.6937];


   C = [ 1,0,0,0,0;

          0,1,0,0,0];

A_bar = [A,zeros(5,2);C,zeros(2,2)];

B_bar= [B;zeros(2,2)];

B1_bar= [zeros(5,2);1 1;1 1];

W=[0 1;-1 0];

V=[2 1; -1 -2];

% m1=1*10^28;

% m2=0.88*10^28;

% W=[0 2.5;-2.5 0];

% V=[3 1;0.1 10];

m1=9*10^10;

m2=9*10^12;

C1=[-10 -20 -2 -20]';
```

```
C2=[0 200 2 20]'
    %%%%%%%%%%%%%%%%%%%%%%%%%%%%
    setlmis([])
P1=lmivar(1,[7,1]);
P2=lmivar(1,[2,1]);
R1z=lmivar(2,[7,2]);% Define R1 transpose
R2z=lmivar(2,[2,5]);% Define R2 transpose
% R1z=lmivar(2,[5,1]);% Define R1 transpose
% R2z=lmivar(2,[5,1]);% Define R2 transpose
% Add LMI term of inequality 1
lmiterm([1 1 1 P1],A_bar,1,'s');
lmiterm([1 1 1 0],C1'*C1);
lmiterm([1 1 1 R1z],1,B_bar','s');
lmiterm([1 1 2 P1],1,B_bar*V);
lmiterm([1 1 3 0],0);
lmiterm([1 1 4 P1],1,B_bar);
lmiterm([1 2 2 P2],1,W,'s');
lmiterm([1 2 2 R2z],1,B*V,'s');
lmiterm([1 2 2 0],C2'*C2);
lmiterm([1 2 4 0],0);
lmiterm([1 3 3 0],-1*m1);
lmiterm([1 3 4 0],0);
lmiterm([1 3 4 0],0);
lmiterm([1 4 4 0],-1*m2);

lmiterm([-2 1 1 P1],1,1);
lmiterm([-3 1 1 P2],1,1);
```

```
% Get the LMI system description

lmisys=getlmis;

[tmin,xfeas]=feasp(lmisys); % LMI solver command

qq=dec2mat(lmisys,xfeas,P1); % converts the output of the solver into the value of the matrix variable P1.

pp=dec2mat(lmisys,xfeas,P2); % converts the output of the solver into the value of the matrix variable P2.

R1z=dec2mat(lmisys,xfeas,R1z); % converts the output of the solver into the value of the matrix variable R1.

R2z=dec2mat(lmisys,xfeas,R2z); % converts the output of the solver into the value of the matrix variable R2.qq;

pp;

R1=R1z';

R2=R2z;

tmin

K=R1*inv(qq)

L=inv(pp)*R2




figure(1)

% % drawing ellipsoid

[x, y, z] = ellipsoid(0, 0, 0, a, b, 0, 100);

surf(x, y, z,'FaceColor', 'red', 'EdgeColor', [0.5 0.5 1],'FaceAlpha',0.1);

xlabel('x');

ylabel('y');
```

```
zlabel('z');
% title(' Reachable set ellipsoid'');
hold on;
plot(yyy1(1,:), yyy1(2,:), 'k', 'LineWidth', 2.5);
% xlabel('${x_1}\left( t \right)$','interpreter','latex','fontsize',16);
%    ylabel('${x_2}\left( t \right)$','interpreter','latex','fontsize',16);
h9=legend(' theorem [3] Ellipsoidal boundary of reachable set',' state trajectory of system'')
set(h9,'Interpreter','latex','fontsize',16);

axis equal;

figure(2)
% Draw the outline of the reachable state
contour(x1_grid, x2_grid, reachable, [0.1 0.1], 'b--', 'LineWidth', 3);
xlabel('${x_1}\left( t \right)$','interpreter','latex','fontsize',16);
ylabel('${x_2}\left( t \right)$','interpreter','latex','fontsize',16);
hold on;
plot(yy1(1,:), yy1(2,:), 'k', 'LineWidth', 3);
% xlabel('${x_1}\left( t \right)$','interpreter','latex','fontsize',16);
%    ylabel('${x_2}\left( t \right)$','interpreter','latex','fontsize',16);
h9=legend(' Under Theorem [3], the reachable state contour of the system',' the state
trajectory of the system. ')
set(h9,'Interpreter','latex','fontsize',16);

figure(3)
% Draw the outline of the reachable state
contour(x1_grid, x2_grid, reachable, [1 1], 'b--', 'LineWidth', 3);
```

```
xlabel('${x_1}\left( t \right)$','interpreter','latex','fontsize',16);

ylabel('${x_2}\left( t \right)$','interpreter','latex','fontsize',16);

hold on;

[x, y, z] = ellipsoid(0, 0, 0, a, b, 0, 100);

surf(x, y, z,'FaceColor', 'red', 'EdgeColor', [0.5 0.5 1],'FaceAlpha',0.1);

hold on;

plot(yyy1(1,:), yyy1(2,:), 'k', 'LineWidth', 3);

% xlabel('${x_1}\left( t \right)$','interpreter','latex','fontsize',16);

%    ylabel('${x_2}\left( t \right)$','interpreter','latex','fontsize',16);

H9=legend ('Contour of reachable state of system under Theorem [3]',' Ellipsoid boundary of reachable set in Theorem [3]',' State trajectory of system')

set(h9,'Interpreter','latex','fontsize',16);


figure(4)

subplot(2,1,1)

  plot(t1,yy1(:,1),'b','linewidth',3);

  xlabel('Time(s)','interpreter','latex','fontsize',16);

    ylabel('${x_1}\left( t \right)$','interpreter','latex','fontsize',16);

% H9=legend ('Theorem [3] Ellipsoid Boundary of Reachable Set',' State Trajectory of System')

% set(h9,'Interpreter','latex','fontsize',16);

subplot(2,1,2)

plot(t1,yy1(:,2),'k','linewidth',3);

  xlabel('Time(s)','interpreter','latex','fontsize',16);

    ylabel('${x_2}\left( t \right)$','interpreter','latex','fontsize',16);
```

```
figure(5)

plot(yy1(1,:), yy1(2,:), 'k', 'LineWidth', 3);

  xlabel('${x_1}\left( t \right)$','interpreter','latex','fontsize',16);

    ylabel('${x_2}\left( t \right)$','interpreter','latex','fontsize',16);

H9=legend ('reachable state contour of the system under Theorem [3]',' state trajectory of the system')

set(h9,'Interpreter','latex','fontsize',16);

hold on;



figure(6)

plot(yy1(1,:), yy1(2,:), 'k', 'LineWidth', 3);

  xlabel('${x_1}\left( t \right)$','interpreter','latex','fontsize',16);

  ylabel('${x_2}\left( t \right)$','interpreter','latex','fontsize',16);

hold on;

plot(0.07*x_rot, 0.08*y_rot, 'b','LineWidth', 3); % Draw the rotated ellipse.

h9=legend('state trajectory','Ellipsoidal boundary of reachable set')

set(h9,'Interpreter','latex','fontsize',16);
```
