# Supplementary material for: Research on reachable set boundary of neutral system with various types of disturbances
Source: PLoS One. 2025 Jan 16;20(1):e0317398. doi: 10.1371/journal.pone.0317398 (PMC11737737; doi:10.1371/journal.pone.0317398)
Supplement: Attachment — Submitted filename: Response-sci.pdf [file pone.0317398.s002.pdf]

Dr Dongmei Xia
Foshan Polytechnic
Foshan, Guangdong Province, 528137, China
E-mail: dreamchaser_edu@163.com

December 11, 2024

Dr. Guojin Qin
Academic Editor PLOS ONE

Ms. No. PONE-D-24-52895

"Research on reachable set boundary of neutral system with various types of disturbances"

Thank you very much for your letter dated Dec 09 2024, informing us of the need for major revisions to our manuscript. Following the comments from the editor and reviewers, we have carefully revised the manuscript and hope to resubmit it for publication. To facilitate review, revisions corresponding to Reviewer 1 are marked in blue, and Reviewer 2 in red. Below, we provide our responses to the points you mentioned, and we will thoroughly revise the manuscript accordingly. **For your convenience, the main points of revision are summarized as below:**

1. We have conducted substantial revisions to address all the reviewers' comments, focusing on improving the clarity and accuracy of the descriptions of our methods, results, and analysis. The manuscript has been restructured to enhance its readability and ensure that the key contributions and findings are effectively communicated.

2. To strengthen the literature review, we have incorporated a broader discussion of recent and relevant studies related to the proposed model. This addition provides better context for our work and highlights its contribution to the existing body of knowledge.

3. We have carefully proofread the manuscript to eliminate typographical and grammatical errors, ensuring a polished and professional presentation that maintains the overall quality and clarity of the paper.

Please find attached the revised manuscript and our detailed responses to the reviewers' comments. Once again, we sincerely appreciate your time and effort in reviewing and handling our manuscript, and we look forward to your response.

Best regards,

Dongmei Xia

Response to Reviewer No. 1:

Ms. No. PONE-D-24-52895

"Research on reachable set boundary of neutral system with various types of disturbances"

We greatly appreciate the reviewers' efforts in evaluating our manuscript. Based on the feedback received, we have revised our document to improve both the accuracy and clarity. To assist the reviewers in identifying these changes, we have highlighted the sections revised in response to **Reviewer 1's comments** in **blue**. Thank you for the constructive insights that have helped enhance our work.

Comment *"This paper is devoted to reachable set boundary of neutral system with various types of disturbances. Some comments are given below:"*

Response:

Thank you for your thoughtful review and constructive comments regarding our manuscript We will address your suggestions by enhancing the theoretical context, refining our simulation studies for broader applicability, and improving the overall clarity of the manuscript to meet the high standards required for impactful publication.

1. **Comment 1:** *"Please enrich your Introduction with extended disturbance-observer-based data-driven control of networked nonlinear systems, output anti-disturbance control of stochastic Markov jump systems with multiple disturbances."*

   Response:

   Thank you for your insightful comments and suggestions regarding our manuscript, We appreciate the opportunity to enhance our paper and address the areas you highlighted.

   We agree that incorporating a discussion on extended disturbance-observer-based data-driven control will provide a more comprehensive understanding of the context and significance of our research. Accordingly, we have added a new paragraph to the Introduction where we discuss recent advancements and methodologies in disturbance-observer-based control for networked nonlinear systems. We have highlighted how these methodologies are pivotal for improving system robustness and performance in the face of network-induced delays and disturbances. We have also expanded our Introduction to include a discussion on output anti-disturbance control for stochastic Markov jump systems. we explore various strategies and recent findings in managing multiple disturbances within these systems, emphasizing the critical role of stochastic modeling in enhancing control efficacy and reliability under uncertain conditions.

   Modification

   ......

   Introduction

   ......

From theoretical research to practical applications, the study of the RS of NTSs exhibit a higher level of complexity and challenge compared to general time-delay systems. In practical fields like control engineering and signal processing, forecasting and managing NTSs pose greater challenges compared to systems that do not feature delays in their derivatives [1, 2, 3, 4, 5]. In [6], Shen and Wang have developed a data-driven control approach for networked nonlinear systems with event-triggered outputs, using an advanced observer to estimate unknown disturbances. They defined triggering conditions for both single and multi-input/output systems based on estimated and actual tracking errors. Sufficient conditions were established to ensure the system's tracking errors remain ultimately bounded, with numerical examples confirming the strategy's effectiveness. In [7], Jiang and Xia investigated the RS estimation for a specific category of Markov jump neutral neural networks, which are influenced by bounded disturbances and exhibit time-varying delays. In [8], in order to find a smaller RS of neutral Markov jump system with disturbance, a new type of LMI is constructed based on Lyapunov function method. Although the research on RS is rarely put forward in neutral Markov jump system, this method can find a more suitable and smaller RS. In addition, an improved LKF is designed to solve the RS ellipsoid of neutral semi-Markov jump systems with disturbances [9]. Shen and Zhang conducted research on anti-disturbance control for Markov jump systems with matched and mismatched disturbances. They developed a composite static output control strategy that includes H_∞ performance to ensure system stability. Additionally, they introduced an integral sliding-mode output (ISMO) control strategy that effectively mitigates disturbances using their upper bounds. The effectiveness of their research was demonstrated through numerical simulations[10]. Different from [11] and [12], Zhang investigated how to estimate the RS for Markov jump bidirectional associative memory neural networks experiencing time-varying delays and bounded disturbances in their inputs. This research aims to understand how these factors influence the network's behavior and stability. Numerical examples show that the simulation results are effective.

......

[6] Mouquan Shen, Xianming Wang, Ju H Park, Yang Yi, and Wei-Wei Che. Extended disturbance-observer-based data-driven control of networked nonlinear systems with event-triggered output. IEEE Transactions on Systems, Man, and Cybernetics: Systems, 53(5):3129-3140, 2022.

[10] Mouquan Shen, Hainan Zhang, Sing Kiong Nguang, and Choon Ki Ahn. H ∞ output anti-disturbance control of stochastic markov jump systems with multiple disturbances. IEEE Transactions on Systems, Man, and Cybernetics: Systems, 51(12):7633-7643, 2020.

We believe that these revisions will make the manuscript stronger and more informative for readers. Thank you again for your constructive feedback.

Please let us know if there are any further modifications you would recommend or require.

2. **Comment 2:** *"If Lemmas 6 and 7 are borrowed from existing results, please give the corresponding citation. Otherwise, please supply the full proof details."*

   Response:

   Thank you for your comments and for giving us the opportunity to clarify the nature of

Lemmas 6 and 7 presented in our manuscript. In order to answer your question, we want to confirm the specific sources of Lemma 6 and Lemma 7. We developed these lemmas to support our research. In order to ensure the clarity and repeatability of our results, we provided the literature sources of these lemmas in the supplementary materials of the manuscript.

Modification

......

Model description and useful lemma:

......

Lemma 5 ([13]): For a vector function $x(t)$ belonging to $\mathbb{R}^n$ that is both continuous and first-order differentiable, consider any matrix $F = \begin{bmatrix} F_1 & F_2 & F_3 & F_4 & F_5 & F_6 & F_7 & F_8 \end{bmatrix}$ and a positive scalar $Z_1 > 0$. Additionally, assume there is a continuous function $h(t)$ where h is a positive real constant. Under these conditions, the following inequality is satisfied when $0 \leqslant h(t) \leqslant h$:

$$-\int_{t-h(t)}^{t} \dot{x}^{\mathrm{T}}(\alpha) Z_1 \dot{x}(\alpha)\mathrm{d}\alpha \leqslant \xi^{\mathrm{T}}(t)\Xi_1\xi(t) + h\xi^{\mathrm{T}}(t)X^{\mathrm{T}}Z_1^{-1}F\xi(t) \tag{1}$$

where

$$\xi(t) = \begin{bmatrix} x^{\mathrm{T}}(t) & \dot{x}^{\mathrm{T}}(t) & x^{\mathrm{T}}(t-h(t)) & x^{\mathrm{T}}(t-h) & \dot{x}^{\mathrm{T}}(t-\tau(t)) & w^{\mathrm{T}}(\sigma(t)) & f_1^{\mathrm{T}} & f_2^{\mathrm{T}} \end{bmatrix}^{\mathrm{T}} \tag{2}$$

$$\Xi_1 = \begin{bmatrix} F_1 + F_1^{\mathrm{T}} & F_2 & F_3 - F_1^{\mathrm{T}} & F_4 & F_5 & F_6 & F_7 & F_8 \\ * & 0 & -F_2^{\mathrm{T}} & 0 & 0 & 0 & 0 & 0 \\ * & * & -F_3 - F_3^{\mathrm{T}} & -F_4 & -F_5 & -F_6 & -F_7 & -F_8 \\ * & * & * & 0 & 0 & 0 & 0 & 0 \\ * & * & * & * & 0 & 0 & 0 & 0 \\ * & * & * & * & * & 0 & 0 & 0 \\ * & * & * & * & * & * & 0 & 0 \\ * & * & * & * & * & * & * & 0 \end{bmatrix}. \tag{3}$$

Applying a technique analogous to that used in Lemma 5 enables us to derive Lemma 6 and Lemma 7.

Lemma 6 ([14]): For a vector function $x(t) \in \mathbb{R}^n$ that is continuous and differentiable, given any matrix $X = \begin{bmatrix} X_1 & X_2 & X_3 & X_4 & X_5 & X_6 & X_7 & X_8 \end{bmatrix}$ and a positive scalar $Z_2 > 0$, along with a continuous function $h(t)$ where $h > 0$, the ensuing inequality is valid provided $0 \leqslant h(t) \leqslant h$:

$$-\int_{t-h}^{t-h(t)} \dot{x}^{\mathrm{T}}(\alpha) Z_2 \dot{x}(\alpha)\mathrm{d}\alpha \leqslant \xi^{\mathrm{T}}(t)\Xi_2\xi(t) + h\xi^{\mathrm{T}}(t)X^{T}Z_2^{-1}F\xi(t) \tag{4}$$

where

$$\xi(t) = \begin{bmatrix} x^{\mathrm{T}}(t) & \dot{x}^{\mathrm{T}}(t) & x^{\mathrm{T}}(t-h(t)) & x^{\mathrm{T}}(t-h) & \dot{x}^{\mathrm{T}}(t-\tau(t)) & w^{\mathrm{T}}(\sigma(t)) & f_1^{\mathrm{T}} & f_2^{\mathrm{T}} \end{bmatrix}^{\mathrm{T}} \tag{5}$$

$$\Xi_2 = \begin{bmatrix} 0 & 0 & X_1^{\mathrm{T}} & -X_1^{\mathrm{T}} & 0 & 0 & 0 & 0 \\ * & 0 & X_2^{\mathrm{T}} & -X_2^{\mathrm{T}} & 0 & 0 & 0 & 0 \\ * & * & X_3 + X_3^{\mathrm{T}} & X_4 - X_3^{\mathrm{T}} & X_5 & X_6 & X_7 & X_8 \\ * & * & * & X_4 - X_3^{\mathrm{T}} & -X_5 & -X_6 & -X_7 & -X_8 \\ * & * & * & * & 0 & 0 & 0 & 0 \\ * & * & * & * & * & 0 & 0 & 0 \\ * & * & * & * & * & * & 0 & 0 \\ * & * & * & * & * & * & * & 0 \end{bmatrix}. \tag{6}$$

Lemma 7 ([15]): Assume $x(t) \in \mathbb{R}^n$ is a continuously differentiable vector function. Take any matrix $Y = \begin{bmatrix} Y_1 & Y_2 & Y_3 & Y_4 & Y_5 & Y_6 & Y_7 & Y_8 \end{bmatrix}$, with each Y_i representing a sub-matrix or vector, and let Z_3 be a positive scalar greater than zero. Furthermore, suppose $h(t)$ is a continuous function satisfying $0 \leq h(t) \leq h$, where h is a fixed positive real number. In this context, the following inequality is satisfied:

$$-\int_{t-h}^{t} \dot{x}^{\mathrm{T}}(\alpha) Z_3 \dot{x}(\alpha) \mathrm{d}\alpha \leqslant \xi^{\mathrm{T}}(t) \Xi_3 \xi(t) + h \xi^{\mathrm{T}}(t) X^{\mathrm{T}} Z_3^{-1} F \xi(t) \tag{7}$$

where

$$\xi(t) = \begin{bmatrix} x^{\mathrm{T}}(t) & \dot{x}^{\mathrm{T}}(t) & x^{\mathrm{T}}(t-h(t)) & x^{\mathrm{T}}(t-h) & \dot{x}^{\mathrm{T}}(t-\tau(t)) & w^{\mathrm{T}}(\sigma(t)) & f_1^{\mathrm{T}} & f_2^{\mathrm{T}} \end{bmatrix}^{\mathrm{T}} \tag{8}$$

$$\Xi_3 = \begin{bmatrix} Y_1 + Y_1^{\mathrm{T}} & Y_2 & Y_3 & -Y_1^{\mathrm{T}} + Y_4 & Y_5 & Y_6 & Y_7 & Y_8 \\ * & 0 & 0 & -Y_2^{\mathrm{T}} & 0 & 0 & 0 & 0 \\ * & * & 0 & -Y_3^{\mathrm{T}} & 0 & 0 & 0 & 0 \\ * & * & * & -Y_4 - Y_4^{\mathrm{T}} & -Y_5 & -Y_6 & -Y_7 & -Y_8 \\ * & * & * & * & 0 & 0 & 0 & 0 \\ * & * & * & * & * & 0 & 0 & 0 \\ * & * & * & * & * & * & 0 & 0 \\ * & * & * & * & * & * & * & 0 \end{bmatrix} \tag{9}$$

......

Once again, thank you for your valuable feedback. Your comments are instrumental in helping us refine and improve our research. We look forward to considering these assumptions and challenges more comprehensively in our future work and conducting more in-depth explorations.

3. **Comment 3:** *“ Please make some necessary comparisons with existing results on theory with some comments.”*

   Response:
   Thank you for your valuable comments on our paper. Regarding your point on ”clarity in terminology and definitions,” we fully understand your concerns and have carefully considered them. Indeed, we used some specialized terms, such as ”partial differential equations” and ”Barrier Lyapunov Function,” without providing comprehensive definitions when they first appear. To improve the readability of the paper and help readers better understand these concepts, we have revised and added clear explanations for these terms.

In the revised version of the paper, we have provided concise and clear definitions in the relevant sections to assist readers in understanding the background and significance of these concepts. For those who are less familiar with these terms, we have also included additional references for further reading and deeper understanding.

Modification

......

Introduction

......

Building on the insights from the aforementioned literature and our investigation into the RS analysis of NTSs, it is evident that analyzing the RS of NTSs is significantly more challenging compared to conventional dynamic systems. This complexity arises from the influence of delay elements, which add layers of difficulty to the theoretical study [5, 16]. Time delays not only impact system stability but can also fundamentally alter system behavior, potentially causing oscillations or instability [17, 18]. In light of these challenges, this paper advances existing research by employing matrix inequality techniques and proposing a novel Lyapunov functional method to identify smaller, more precise RS boundaries for NTSs. The primary contributions of this work are summarized as follows.

0.1 First of all, many practical engineering systems inherently exhibit delay characteristics. The study of NTSs plays a crucial role in designing stable and robust control systems by providing theoretical insights and methodological solutions to effectively address delay-related challenges, making it highly significant for real-world applications.

0.2 Furthermore, this work delves into the determination of elliptical boundaries for the RS in NTSs. By developing innovative Lyapunov functionals and leveraging advanced matrix inequality techniques, several methodologies were proposed to identify minimal elliptical boundaries of the RS. These contributions enhance both the theoretical foundation and practical applicability of RS analysis in NTSs, which are essential for progress in control design, system optimization, and safety assurance.

0.3 Finally, compared to other research efforts on the RS of NTSs (e.g., [19], [20]), numerical simulations using MATLAB/SIMULINK have demonstrated that the proposed approach not only effectively encompasses all system trajectories but also achieves smaller boundaries for the ellipsoidal RS, underscoring its efficiency and accuracy.

......

Modification

......

Simulation verification

......

Furthermore, Figure 1 contrasts the state trajectories with a constant disturbance of $w(t) = 0.55$ against ellipsoidal RS boundaries based on Theorem 1 from this study, as well as those

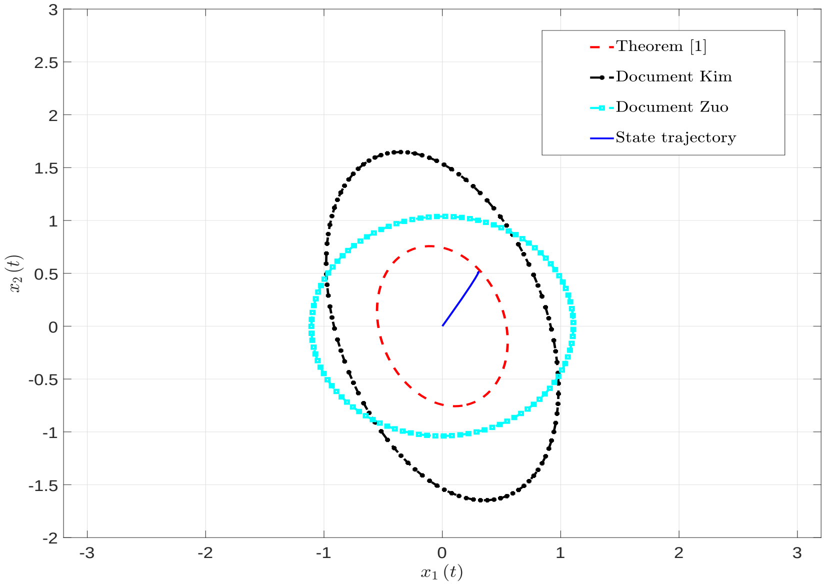

Figure 1: The RS system state trajectory diagram of different methods under constant disturbance.

established using the Kim method (see Reference [19]) and the Zuo method (see Reference [20]). The ellipsoidal boundaries for the RS generated by the method described in Theorem 1 are notably smaller than those determined by the prior methods.

......

Finally, Figure 2 compares the RS of the nonlinear NTSs using the proposed method against the methods referenced in [20] and [19]. This comparison shows that the proposed method not only ensures complete containment of the system state trajectory but also achieves a smaller RS compared to existing methods.

The authors wish to extend their heartfelt gratitude to the referee for their valuable and insightful feedback. The constructive yet critical comments provided by the referee have been instrumental in refining both the quality and the clarity of this work. Their thoughtful suggestions and detailed efforts have significantly contributed to enhancing the overall presentation and rigor of the paper, for which we are deeply appreciative.

References

[1] Pin-Lin Liu. Improved results on delay-interval-dependent robust stability criteria for uncertain neutral-type systems with time-varying delays. *Isa Transactions*, 60:53–66, 2016.

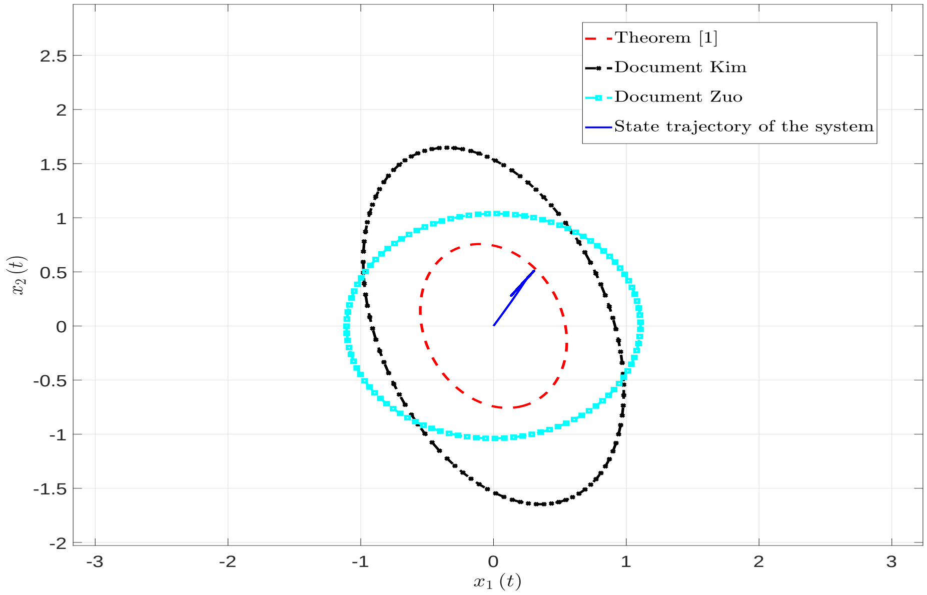

Figure 2: The trajectory diagrams of the RS system state for various methods under time-varying disturbance.

[2] Li Wan, Qinghua Zhou, Hongbo Fu, and Qunjiao Zhang. Exponential stability of hopfield neural networks of neutral type with multiple time-varying delays. *AIMS Math*, 6(8):8030–8043, 2021.

[3] Xiaohui Duan, Fangping Tang, and Wenyong Duan. Improved robust stability criteria for uncertain linear neutral-type systems via novel lyapunov-krasovskii functional. *Asian Journal of Control*, 22(2):976–987, 2020.

[4] Wim Michiels and Tomáš Vyhlídal. An eigenvalue based approach for the stabilization of linear time-delay systems of neutral type. *Automatica*, 41(6):991–998, 2005.

[5] Zahra Sadat Aghayan, Alireza Alfi, and JA Tenreiro Machado. Robust stability of uncertain fractional order systems of neutral type with distributed delays and control input saturation. *ISA transactions*, 111:144–155, 2021.

[6] Mouquan Shen, Xianming Wang, Ju H Park, Yang Yi, and Wei-Wei Che. Extended disturbance-observer-based data-driven control of networked nonlinear systems with event-triggered output. *IEEE Transactions on Systems, Man, and Cybernetics: Systems*, 53(5):3129–3140, 2022.

[7] Xiangli Jiang, Guihua Xia, Zhiguang Feng, Zhengyi Jiang, and Jianbin Qiu. Reachable set estimation for markovian jump neutral-type neural networks with time-varying delays. *IEEE Transactions on Cybernetics*, 52(2):1150–1163, 2020.

[8] Changchun Shen, Shouwei Zhou, Hongyong Deng, et al. The no-ellipsoidal bound of reachable sets for neutral markovian jump systems with disturbances. *Journal of Applied Mathematics and Physics*, 8(05):799, 2020.

[9] Xipan Zhang, Changchun Shen, and Dingju Xu. Reachable set estimation for neutral semi-markovian jump systems with time-varying delay. *AIMS Mathematics*, 9(4):8043–8062, 2024.

[10] Mouquan Shen, Hainan Zhang, Sing Kiong Nguang, and Choon Ki Ahn. H∞ output anti-disturbance control of stochastic markov jump systems with multiple disturbances. *IEEE Transactions on Systems, Man, and Cybernetics: Systems*, 51(12):7633–7643, 2020.

[11] Zhiguang Feng, Wei Xing Zheng, and Ligang Wu. Reachable set estimation of t–s fuzzy systems with time-varying delay. *IEEE Transactions on Fuzzy Systems*, 25(4):878–891, 2016.

[12] Zhiqiang Zuo, Yinping Chen, Yijing Wang, Daniel WC Ho, Michael ZQ Chen, and Hongchao Li. A note on reachable set bounding for delayed systems with polytopic uncertainties. *Journal of the Franklin Institute*, 350(7):1827–1835, 2013.

[13] Jack K Hale and Kenneth R Meyer. *A class of functional equations of neutral type*, volume 76. American Mathematical Society Providence, RI, USA, 1967.

[14] Changchun Shen and Shouming Zhong. The ellipsoidal bound of reachable sets for linear neutral systems with disturbances. *Journal of the Franklin Institute*, 348(9):2570–2585, 2011.

[15] Liang Zhang, Xudong Zhao, and Ning Zhao. Real-time reachable set control for neutral singular markov jump systems with mixed delays. *IEEE Transactions on Circuits and Systems II: Express Briefs*, 69(3):1367–1371, 2021.

[16] Zahra Sadat Aghayan, Alireza Alfi, Yashar Mousavi, and Afef Fekih. Stability analysis of a class of variable fractional-order uncertain neutral-type systems with time-varying delay. *Journal of the Franklin Institute*, 360(14):10517–10535, 2023.

[17] Gerson Portilla, Irina V Alexandrova, and Sabine Mondié. Stability tests for neutral-type delay systems: A delay lyapunov matrix and piecewise linear approximation of the functional argument approach. *International Journal of Robust and Nonlinear Control*, 2024.

[18] Leonid Berezansky, Josef Diblík, Zdeněk Svoboda, and Zdeněk Šmarda. Exponential stability criteria for linear neutral systems with applications to neural networks of neutral type. *Journal of the Franklin Institute*, 360(1):301–326, 2023.

[19] Jin-Hoon Kim. Improved ellipsoidal bound of reachable sets for time-delayed linear systems with disturbances. *Automatica*, 44(11):2940–2943, 2008.

[20] Zhiqiang Zuo, Daniel WC Ho, and Yijing Wang. Reachable set bounding for delayed systems with polytopic uncertainties: the maximal lyapunov–krasovskii functional approach. *Automatica*, 46(5):949–952, 2010.

[21] Chunbin Qin, Tianzeng Zhu, Kaijun Jiang, and Yinliang Wu. Integral reinforcement learning-based dynamic event-triggered safety control for multiplayer stackelberg–nash games with time-varying state constraints. *Engineering Applications of Artificial Intelligence*, 133:108317, 2024.

[22] Chunbin Qin, Ziyang Shang, Zhongwei Zhang, Dehua Zhang, and Jishi Zhang. Parallel learning-based security robust tracking control for nonlinear systems with uncertainties: An event-triggered design. *Engineering Applications of Artificial Intelligence*, 133:108077, 2024.

[23] Chunbin Qin, Xiaopeng Qiao, Jinguang Wang, Dehua Zhang, Yandong Hou, and Shaolin Hu. Barrier-critic adaptive robust control of nonzero-sum differential games for uncertain nonlinear systems with state constraints. *IEEE Transactions on Systems, Man, and Cybernetics: Systems*, 2023.

Response to Reviewer No. 2:

Ms. No. PONE-D-24-52895

"Research on reachable set boundary of neutral system with various types of disturbances"

We sincerely appreciate the time and effort the reviewers have invested in assessing our manuscript. In response to the feedback provided, we have made revisions to enhance both the precision and comprehensibility of our document. To facilitate the reviewers' assessment of these changes, we have marked the sections modified in response to **Reviewer 2's comments** in **red**. We are grateful for the valuable insights that have significantly improved our manuscript.

"In this paper, the reachable set for NTSs is studied by using the Lyapunov functional conjunction with various matrix inequality techniques. And the safe, efficient, and minimized boundaries of the RS for NTSs is demonstrated via an example. In addition, there are quite a few problems in the paper. Specific suggestions for improvement of the manuscript."

Response:
Thank you for your valuable feedback and the opportunity to enhance our manuscript. We appreciate your insights on the use of Lyapunov functional in conjunction with various matrix inequality techniques to study the reachable set (RS) for nonlinear time-delay systems (NTSs). We are also grateful for your recognition of our efforts to demonstrate the safe, efficient, and minimized boundaries of the RS for NTSs through an example. Regarding the issues you have raised, we offer the following responses and clarifications:

1. **Comment 1:** *"In the title, various types of disturbances are shown, but how to dealt with them is not explained in the paper?"*

   Response:

   Thank you for your insightful comment regarding the treatment of various types of disturbances as mentioned in the title of our paper. We appreciate the opportunity to clarify this aspect, which may have not been sufficiently detailed in our manuscript.

   In our study, we broadly categorize disturbances into linear and nonlinear types based on their characteristics and impact on the system dynamics. Here is how we address each type:

   Linear Disturbances: These are treated through the inclusion of specific matrix terms in our Lyapunov function analysis. The terms involving $w(t)$ in the Lyapunov derivative $\dot{V}(t)$ directly account for linear disturbances, with energy bounded by $w^{\mathrm{T}}(t)w(t) \leqslant 1$. This approach allows us to stabilize the system against predictable variations that can be mathematically modeled as linear perturbations.

   Nonlinear Disturbances: For disturbances that exhibit nonlinear behavior, we implement the S-procedure to integrate the impact of such disturbances into our stability analysis. The nonlinear terms $f_1(x, x(t))$ and $f_2(x, x(t-h(t)))$ are modeled within the

Lyapunov function framework to ensure robustness against more complex dynamics that cannot be linearized. The parameters ε_1 and ε_2 in our matrix inequalities are adjusted to optimize the system's response to these disturbances, as detailed in Equations (70) and (71) of our manuscript.

Furthermore, we acknowledge that the detailed mechanisms dealing with each specific type of disturbance could have been more explicitly stated in the paper. We are currently revising the manuscript to include a more detailed discussion on this topic, along with additional examples illustrating the application of our approach to different disturbance scenarios.

Modification

......

From the analysis discussed, the NTS examined in this section can be characterized as

$$\begin{cases} \dot{x}(t) - C\dot{x}(t-\tau) = & A_1 x(t) + A_2 x(t-h(t)) + Bw(t) \\ & + D_1 f_1(t, x(t)) + D_2 f_2(t, x(t-h(t))), \\ x(t_0+\theta) = \varphi(\theta), \forall \theta \in [-\tau^*, 0]. \end{cases} \tag{10}$$

where $x(t) \in \mathbb{R}^n$ signifies the state vector of the system, $w(t) \in \mathbb{R}^l$ corresponds to disturbances, and $\varphi(\cdot)$ is a differentiable function defining initial conditions. Here, τ is a constant delay used specifically in the term $\dot{x}(t-\tau)$, and $h(t)$ is a time-varying delay affecting the state term $x(t-h(t))$ and adds additional dynamism to the system's response to past states. Both the neutral-type delay and the disturbances are subject to the following conditions:

$$0 \leqslant h(t) \leqslant h \leqslant +\infty \tag{11}$$

$$\dot{h}(t) \leqslant h_d \leqslant 1 \tag{12}$$

$$w^{\mathrm{T}}(t)w(t) \leqslant 1 \tag{13}$$

In this section, we address the RS boundary issue for the aforementioned NTSs, choose the suitable Lyapunov functional, and present the following theorem:

Theorem 1: For the neutral system Eq.(25), if there exist real matrices $X = [X_1, X_2, X_3, X_4, X_5, X_6, X_7, X_8$ $Y = [Y_1, Y_2, Y_3, Y_4, Y_5, Y_6, Y_7, Y_8]$, and $Z = [Z_1, Z_2, Z_3, Z_4, Z_5, Z_6, Z_7, Z_8]$, along with the matrices Q_{12}, P_2, and P_3, and symmetric matrices Q_{11}, Q_{22}, Q_{12}, $P_1 > 0$, $R_1 > 0$, $R_2 > 0$, $R_3 > 0$, $S_1 \geqslant 0$, $S_2 > 0$, $S_3 > 0$, and $G > 0$, as well as a scalar $\alpha > 0$, such that the following linear matrix inequalities are satisfied:

$$\begin{bmatrix} \Phi & hF^{\mathrm{T}} & hX^{\mathrm{T}} & hY^{\mathrm{T}} \\ * & -\mathrm{e}^{-\alpha h}S_1 & 0 & 0 \\ * & * & -\mathrm{e}^{-\alpha h}S_2 & 0 \\ * & * & * & -\mathrm{e}^{-\alpha h}S_3 \end{bmatrix} \leqslant 0 \tag{14}$$

$$\begin{bmatrix} Q_{11} & Q_{12} \\ Q_{12}^{\mathrm{T}} & Q_{22} \end{bmatrix} \geqslant 0, \tag{15}$$

$$\begin{bmatrix} X & Y \\ Y^{\mathrm{T}} & Z + \frac{1}{\tau}\mathrm{e}^{-\alpha\tau}R \end{bmatrix} \geqslant 0 \tag{16}$$

where

$$\Phi = \begin{bmatrix} \Phi_{11} & \Phi_{12} & \Phi_{13} & \Phi_{14} & \Phi_{15} & \Phi_{16} & \Phi_{17} & \Phi_{18} \\ * & \Phi_{22} & \Phi_{23} & -hX_2^{\mathrm{T}} - hY_2^{\mathrm{T}} & P_3^{\mathrm{T}}C & P_3^{\mathrm{T}}B & P_3^{\mathrm{T}}D_1 & P_3^{\mathrm{T}}D_2 \\ * & * & \Phi_{33} & \Phi_{34} & \Phi_{35} & \Phi_{36} & \Phi_{37} & \Phi_{38} \\ * & * & * & \Phi_{44} & \Phi_{45} & \Phi_{46} & \Phi_{47} & \Phi_{48} \\ * & * & * & * & -\mathrm{e}^{-\alpha\tau}R_4 & 0 & 0 & 0 \\ * & * & * & * & * & -\frac{\alpha}{w_m^2 I} & 0 & 0 \\ * & * & * & * & * & * & -\varepsilon_1 I & 0 \\ * & * & * & * & * & * & * & -\varepsilon_2 I \end{bmatrix}, \tag{17}$$

Based on condition Eq.(6), Eq.(7) and using the S-procedure, we can derive the following inequality:

$$\begin{aligned} &\dot{V}(t) + \alpha V(t) - \frac{\alpha}{w_m^2} w^{\mathrm{T}}(t)w(t) \\ &\leqslant \xi^{\mathrm{T}}(t)\left[\hat{\Phi} + h^2\mathrm{e}^{\alpha h}F^{\mathrm{T}}S_1^{-1}F + h^2\mathrm{e}^{\alpha h}X^{\mathrm{T}}S_2^{-1}X + h^2\mathrm{e}^{\alpha h}Y^{\mathrm{T}}S_3^{-1}Y\right]\xi(t) \\ &\quad + \varepsilon_1\left[\lambda_1^2 x^{\mathrm{T}}(t)x(t) - f_1^{\mathrm{T}}(x, x(t))f_1(x, x(t))f_1(x, x(t))\right] \\ &\varepsilon_2\left[\lambda_2^2 x^{\mathrm{T}}(t-h(t))x(t-h(t)) - f_2^{\mathrm{T}}(x, x(t-h(t)))f_2(x, x(t-h(t)))\right]. \end{aligned} \tag{18}$$

From the matrix inequalities Eq.(14) and Eq.(15), we can derive:

$$\begin{aligned} &\dot{V}(t) + \alpha V(t) - \frac{\alpha}{w_m^2} w^{\mathrm{T}}(t)w(t) \\ &\leqslant \xi^{\mathrm{T}}(t)\left[\hat{\Phi} + h^2\mathrm{e}^{\alpha h}F^{\mathrm{T}}S_1^{-1}F + h^2\mathrm{e}^{\alpha h}X^{\mathrm{T}}S_2^{-1}X + h^2\mathrm{e}^{\alpha h}Y^{\mathrm{T}}S_3^{-1}Y\right]\xi(t) \\ &\quad + \varepsilon_1\left[\lambda_1^2 x^{\mathrm{T}}(t)x(t) - f_1^{\mathrm{T}}(x, x(t))f_1(x, x(t))\right] \\ &\quad + \varepsilon_2\left[\lambda_2^2 x^{\mathrm{T}}(t-h(t))x(t-h(t)) - f_2^{\mathrm{T}}(x, x(t-h(t)))f_2(x, x(t-h(t)))\right] \leqslant 0. \end{aligned} \tag{19}$$

Thus, we can conclude:

$$\dot{V}(t) + \alpha V(t) - \frac{\alpha}{w_m^2} w^{\mathrm{T}}(t)w(t) \leqslant 0. \tag{20}$$

Based on Lemma 3, we can determine

$$V(t) = V_1(t) + V_2(t) + V_3(t) + V_4(t) \leqslant 1 \tag{21}$$

Furthermore, inequality Eq.(29) implies $V_2(t) + V_3(t) + V_4(t) > 0$, hence:

$$V_1(t) = x^{\mathrm{T}}(t)P_1 x(t) \leqslant 1 \tag{22}$$

is valid. With this, the theorem is proven.

We hope this clarification addresses your concern, and we thank you again for helping us improve the quality and impact of our work.

2. **Comment 2:** *"In the article, the system includes the problem of time delay, but The issue of time delay was not considered in the abstract."*

   Response:

   Thank you for your astute observation regarding the omission of the time delay issue in the abstract of our manuscript.

   You are correct in noting that while our study extensively addresses the impact of time-varying delays within the framework of neutral-type systems (NTSs), this critical aspect was not highlighted in the abstract. Time delays, both constant and time-varying, are indeed fundamental to our analysis and are integral to the development of the minimized ellipsoidal boundaries of the reachable sets (RS) discussed in the paper.

   The omission was an oversight, and we appreciate your pointing it out. To rectify this, we propose to revise the abstract to explicitly mention the role of time delays. A suggested addition to the abstract might read as follows:

   Modification

   ......

   Abstract

   ......

   This study delves into neutral-type systems (NTSs), emphasizing the critical role of defining precise reachable set (RS) boundaries for safe and efficient system design and operation. The investigation notably addresses the challenges posed by time-varying delays, applying Lyapunov's direct method alongside advanced matrix inequality techniques to identify minimized and more accurate ellipsoidal boundaries of the RS in NTSs influenced by bounded and nonlinear disturbances. Our findings, verified through numerical simulations and comparisons with existing literature, demonstrate enhanced control and management capabilities for complex systems, thus underscoring the substantial theoretical and practical value of incorporating delay elements in NTSs.

   We believe that this revision will better encapsulate the study's scope and contributions, aligning the abstract with the detailed discussions presented in the manuscript. We will ensure that the revised abstract accurately reflects the content of the paper and highlights all key aspects, including the treatment of time delays.

   Thank you once again for your constructive comments, which have undoubtedly helped in enhancing the clarity and completeness of our manuscript.

3. **Comment 3:** *"The abstract is not an introduction, please revise the abstract carefully. There are too many things in the introduction that are not relevant to the content of this article. There is almost no background to this paper, just a mention of the goals that the proposed model needs to achieve. Moreover, it provides too little introduction to relevant studies on the detection methods. Some ideas like Integral reinforcement learning-based dynamic event-triggered safety control for multiplayer Stackelberg–Nash games with time-varying state constraints, Engineering Applications of Artificial Intelligence; Parallel*

learning-based security robust tracking control for nonlinear systems with uncertainties: An event-triggered design, , Engineering Applications of Artificial Intelligence; Barrier-critic adaptive robust control of nonzero-sum differential games for uncertain nonlinear systems with state constraints,IEEE Transactions on Systems, Man, and Cybernetics: Systems. can obviously enhance the article's quality and impact."

Response:

Thank you for your detailed and constructive feedback on our manuscript. We appreciate the time and effort you have dedicated to reviewing our work. Below, we address your concerns and describe the corresponding revisions we plan to implement.

Comment on the Abstract:

You mentioned that the abstract reads more like an introduction and needs careful revision. We acknowledge this observation and agree that the abstract should focus on a concise summary of the paper's scope, methodology, and findings rather than introductory remarks. Proposed Revision: We will revise the abstract to remove introductory content and reframe it to include the following key elements:

A brief background and motivation.

A concise description of the proposed approach.

A summary of the main results and contributions.

We will incorporate discussions of the following suggested references:

[21] Qin C, Zhu T, Jiang K, et al. Integral reinforcement learning-based dynamic event-triggered safety control for multiplayer Stackelberg–Nash games with time-varying state constraints[J]. Engineering Applications of Artificial Intelligence, 2024, 133: 108317.

[22] Qin C, Shang Z, Zhang Z, et al. Parallel learning-based security robust tracking control for nonlinear systems with uncertainties: An event-triggered design[J]. Engineering Applications of Artificial Intelligence, 2024, 133: 108077.

[23] Qin C, Qiao X, Wang J, et al. Barrier-critic adaptive robust control of nonzero-sum differential games for uncertain nonlinear systems with state constraints[J]. IEEE Transactions on Systems, Man, and Cybernetics: Systems, 2023.

These references will be used to strengthen the literature review, highlight the novelty of our approach, and position our work within the broader research landscape.

Modification

......

Introduction

......

The investigation of the RS for NTSs presents more complex and challenging scenarios than typical time-delay systems, both in theoretical exploration and practical application. In areas such as control engineering and signal processing, predicting and managing the behavior of NTSs is notably more challenging than dealing with systems lacking delays in their derivatives[1, 2, 3, 4, 5]. In [6], Shen and Wang have developed a data-driven control

approach for networked nonlinear systems with event-triggered outputs, using an advanced observer to estimate unknown disturbances. They defined triggering conditions for both single and multi-input/output systems based on estimated and actual tracking errors. Sufficient conditions were established to ensure the system's tracking errors remain ultimately bounded, with numerical examples confirming the strategy's effectiveness. In [22], Qin and colleagues developed an adaptive safety tracking control strategy suitable for nonlinear systems with both matched and mismatched uncertainties. This strategy optimizes system performance through control barrier functions (CBF) and event-triggering mechanisms, uses adaptive neural networks to precisely approximate solutions, and employs Lyapunov theory to ensure the uniform ultimate boundedness (UUB) of system errors. Simulation results validate the effectiveness of this method. In [7], Jiang and Xia investigated the RS estimation for a specific category of Markov jump neutral neural networks, which are influenced by bounded disturbances and exhibit time-varying delays. With the help of delay segmentation method, numerical simulation confirmed the correctness of theoretical analysis. Similarly intriguing as the work in Reference [22], Qin and Qiao et al. have developed an adaptive stabilization strategy based on the CBF for nonzero-sum differential games in uncertain nonlinear systems with state constraints. This strategy takes into account random disturbances and uncertainties in the control input matrix, utilizing a nominal system and custom cost functions to effectively transform the robust control problem of multiple players into an optimal control problem. By employing Lyapunov theory, it ensures that the system states and neural network weights are uniformly UUB, with the effectiveness of the approach validated through two simulation examples[23].

......

Different from [11] and [12], Zhang investigated how to estimate the RS for Markov jump bidirectional associative memory neural networks experiencing time-varying delays and bounded disturbances in their inputs. This research aims to understand how these factors influence the network's behavior and stability. Numerical examples show that the simulation results are effective. In [21], Qin and Zhu developed a new dynamic event-triggered safety control strategy based on integral reinforcement learning, specifically designed to address multi-player Stackelberg-Nash game problems in continuous-time nonlinear systems with time-varying state constraints. They simplified the constrained problem by integrating innovative barrier functions with state transformation techniques, and implemented a dynamic event-triggering mechanism along with a single critic neural network to ensure system stability and effective control of errors.

We believe these revisions will address your concerns and significantly improve the quality and clarity of the manuscript. Thank you again for your valuable feedback, which has been instrumental in enhancing our work.

4. **Comment 4:** *"In addition, the main contributions described in this article are vague. The main contributions described in this article does not describe how to handle disturbances."*

   Response:

   Thank you for your feedback and for highlighting areas in our manuscript where the description of the main contributions and the handling of disturbances may have been

unclear. We appreciate the opportunity to clarify these aspects, which are central to our research.

We realize that our initial presentation might not have explicitly articulated the novel aspects of our research. To address this, we have revised the manuscript to clearly define and highlight our main contributions, which include:

Developing a comprehensive mathematical model for neutral-type systems (NTS) that incorporates both time-varying neutral-type and discrete delays, providing a framework that captures the dynamic interactions within these systems more accurately than existing models. Introducing an advanced control framework that uses matrix inequalities and Lyapunov stability theory to ensure robust stability and control in the presence of nonlinear disturbances and dynamically changing system states.

Handling of Disturbances: We acknowledge that our discussion on how disturbances are handled in the system might not have been sufficiently detailed. In our revised manuscript, we have expanded this section to better illustrate how disturbances are integrated into our model and controlled through our proposed framework:

The disturbances $w(t)$ are explicitly considered within our mathematical model and are constrained by power, which ensures that their influence is bounded and manageable. This is critical for maintaining system stability under real-world conditions.

We utilize a robust control approach that incorporates disturbances into the system's feedback mechanism through a dynamic event-triggered control strategy. This strategy adjusts the control actions based on the magnitude of disturbances, ensuring that the system's performance remains optimal even in the face of unexpected changes.

To further substantiate our contributions and the effectiveness of our disturbance handling techniques, we have included additional simulation examples that demonstrate the resilience of our approach against high-intensity disturbances across various scenarios.

We hope these revisions and clarifications address your concerns and illustrate the value and novelty of our work more clearly. We thank you again for your constructive comments, which have undoubtedly helped improve the quality and impact of our manuscript.

5. **Comment 5:** *"How to get the equation (2)?'*

   Response:

   Thank you for your inquiry regarding the derivation of Equation (2) from our manuscript. I appreciate the opportunity to clarify the formulation and the underlying assumptions that lead to this representation of the neutral-type system (NTS) with time delays.

   Equation (2) is derived based on the general dynamics of a neutral-type delay differential system, which inherently includes state delays and derivative terms influenced by past states. The specific form of Equation (2) is a result of expanding the traditional delay differential equation to include system dynamics affected by time-varying delays, disturbances, and nonlinear feedback terms. Here's a step-by-step explanation:

   General Delay Differential Equation: The basic form of a neutral-type delay differential equation (DDE) is given by Equation (1):

$$\dot{x}(t) = f(x(t), x(t-\tau), \dot{x}(t-\tau)) \tag{23}$$

This represents the rate of change of the state $x(t)$ depending on its current state, its state at some past time $t-\tau$, and the rate of change at that past time.

System Matrices and Delay Terms: In our model, A_1 and A_2 represent the linear state feedback matrices corresponding to the current state and the state at a delayed time $t-$ $h(t)$, respectively. This helps in modeling systems where the delay $h(t)$ can vary over time and is different from τ, the fixed delay affecting the derivative term.

Disturbance Vector: The term $Bw(t)$ models external disturbances affecting the system, where B is a matrix that scales the disturbance vector $w(t)$.

Nonlinear Feedback Functions: The functions $D_1 f_1(t, x(t))$ and $D_2 f_2(t, x(t-h(t)))$ are introduced to capture nonlinear dependencies of the system's dynamics on the current and delayed states, scaled by the matrices D_1 and D_2.

Derivation of the Modified Equation: To form Equation (2), we start with the standard delay differential equation and incorporate terms that model the influence of varying delays, disturbances, and nonlinear effects as described. The derivative of the state $\dot{x}(t)$ is adjusted by subtracting the term $C\dot{x}(t-\tau)$ to incorporate the effect of the past rate of change, modulated by matrix C, reflecting the neutral-type characteristics of the delay.

The term $x(t_0 + \theta) = \varphi(\theta)$ specifies the initial state of the system for all θ within the interval $[-\tau^*, 0]$, where τ^* is the maximum delay encountered in the system, ensuring all necessary initial conditions are defined for the solution of the DDE.

We hope this detailed explanation clarifies the derivation and correctness of Equation (2) as it pertains to our study of NTS with variable time delays and nonlinearities. Please let us know if further details are needed.

6. **Comment 6:** *"In the equation (2),   (t) is a time-varying delay?'*

   Response:

   Thank you for your insightful question regarding the nature of the delay represented in Equation (2) of our manuscript. I apologize for any confusion that may have arisen from the notation used, and I appreciate the opportunity to clarify this point.

   In Equation (2), τ represents a fixed delay affecting the derivative term $\dot{x}(t-\tau)$. This is a constant value, meaning it does not vary with time. The equation is intended to model the impact of this fixed delay on the dynamics of the neutral-type system (NTS). Here, τ is used to capture the delay between the current state and a previous state, which influences the current rate of change of the system state.

   However, the equation also incorporates $h(t)$, which is indeed a time-varying delay, affecting different aspects of the system dynamics. The term $x(t-h(t))$ represents the system state at a past time where $h(t)$ varies over time, providing a model for more complex dynamic interactions within the system that can change as conditions evolve.

   To summarize:

τ is a constant delay used specifically in the term $\dot{x}(t-\tau)$.

$h(t)$ is a time-varying delay affecting the state term $x(t-h(t))$ and adds additional dynamism to the system's response to past states.

Modification

......

Consider the general representation of a NTS, characterized by the following delay differential equation:

$$\dot{x}(t) = f(x(t), x(t-\tau), \dot{x}(t-\tau)) \tag{24}$$

where, $x(t)$ denotes the system's state at time t, $\dot{x}(t)$ indicates the rate of change of the state variable over time, τ represents the delay, and f is a function that dictates the system's evolution through time.

From the analysis discussed, the NTS examined in this section can be characterized as

$$\begin{cases} \dot{x}(t) - C\dot{x}(t-\tau) = A_1 x(t) + A_2 x(t-h(t)) + Bw(t) \\ \qquad + D_1 f_1(t, x(t)) + D_2 f_2(t, x(t-h(t))), \\ x(t_0+\theta) = \varphi(\theta), \forall \theta \in [-\tau^*, 0]. \end{cases} \tag{25}$$

where, $x(t) \in \mathbb{R}^n$ signifies the state vector of the system, $w(t) \in \mathbb{R}^l$ corresponds to disturbances, and $\varphi(\cdot)$ is a differentiable function defining initial conditions. Here, τ is a constant delay used specifically in the term $\dot{x}(t-\tau)$, and $h(t)$ is a time-varying delay affecting the state term $x(t-h(t))$ and adds additional dynamism to the system's response to past states. Both the neutral-type delay and the disturbances are subject to the following conditions:

$$0 \leqslant h(t) \leqslant h \leqslant +\infty \tag{26}$$

$$\dot{h}(t) \leqslant h_d \leqslant 1 \tag{27}$$

$$w^{\mathrm{T}}(t) w(t) \leqslant 1 \tag{28}$$

I hope this clarification helps, and I am happy to provide further details if needed. Thank you again for your attention to detail and for helping us improve the clarity and accuracy of our work.

7. **Comment 7:** *"In Lyapunov functional (51), Where is the innovation reflected in the equation (51)?'*

Response:

Thank you for your question regarding the innovation in the Lyapunov functional described in our manuscript. I appreciate the opportunity to elucidate how this functional is uniquely crafted to cater to the dynamics and complexities of the neutral-type system (NTS) with time-varying delays, as outlined in equation (2).

The Lyapunov functional $V(t) = V_1(t) + V_2(t) + V_3(t) + V_4(t)$ is innovatively designed to capture and effectively manage the stability challenges posed by the NTS's delay characteristics and its system dynamics. Here are the innovative aspects of each component of the Lyapunov functional:

Proof: To obtain a smaller RS boundary for the system Eq.(25), we choose the following Lyapunov functional as

$$V(t) = V_1(t) + V_2(t) + V_3(t) + V_4(t) \tag{29}$$

where

$$V_1(t) = \begin{bmatrix} x^{\mathrm{T}}(t) & \dot{x}^{\mathrm{T}}(t) \end{bmatrix} \begin{bmatrix} I & 0 \\ 0 & 0 \end{bmatrix} \begin{bmatrix} P_1 & 0 \\ P_2 & P_3 \end{bmatrix} \begin{bmatrix} x(t) \\ \dot{x}(t) \end{bmatrix} \tag{30}$$

$$\begin{aligned} V_2(t) = & \int_{t-h(t)}^{t} \mathrm{e}^{\alpha(s-t)} x^{\mathrm{T}}(s) R_1 x(s) \mathrm{d}s + \int_{t-h}^{t} \mathrm{e}^{\alpha(s-t)} x^{\mathrm{T}}(s) R_2 x(s) \mathrm{d}s \\ & + \int_{t-h}^{t-h(t)} \mathrm{e}^{\alpha(s-t)} x^{\mathrm{T}}(s) R_3 x(s) \mathrm{d}s + \int_{t-\tau}^{t} \mathrm{e}^{\alpha(s-t)} \dot{x}^{\mathrm{T}}(s) R_4 \dot{x}(s) \mathrm{d}s \end{aligned} \tag{31}$$

$$V_3(t) = h \int_{-h}^{0} \mathrm{d}\theta \int_{t+\theta}^{t} \mathrm{e}^{\alpha(s-t)} \dot{x}^{\mathrm{T}}(s) \left(S_1 + S_2 + S_3\right) \dot{x}(s) \mathrm{d}s \tag{32}$$

$$\begin{aligned} V_4(t) = & \int_{0}^{t} \mathrm{d}\theta \int_{\theta-h(\theta)}^{\theta} \mathrm{e}^{\alpha(\theta-t)} \begin{bmatrix} \dot{x}^{\mathrm{T}}(s) & x^{\mathrm{T}}(\theta-h(\theta)) \end{bmatrix} \begin{bmatrix} Q_{11} & Q_{12} \\ Q_{12}^{\mathrm{T}} & Q_{22} \end{bmatrix} \begin{bmatrix} \dot{x}(s) \\ x(\theta-h(\theta)) \end{bmatrix} \mathrm{d}s \\ & + \mathrm{e}^{\alpha h} \int_{0}^{t} \mathrm{d}\theta \int_{\theta-h(\theta)}^{\theta} \mathrm{e}^{\alpha(s-t)} \dot{x}^{\mathrm{T}}(s) Q_{11} \dot{x}(s) \mathrm{d}s \end{aligned} \tag{33}$$

Matrix Structuring in $V_1(t)$:

The matrix configuration within $V_1(t)$, featuring the block diagonal matrix and dynamic matrices P_1, P_2, and P_3, is tailored to encapsulate both the current state $x(t)$ and its derivative $\dot{x}(t)$. This dual consideration is crucial for handling the neutral-type behavior of the system, where both the state and its rate are significant.

Incorporation of Time-Varying Delays in $V_2(t)$ and $V_3(t)$:

$V_2(t)$ and $V_3(t)$ incorporate integrals over past states with weights that exponentially decay, reflecting the influence of the state at past time intervals influenced by the delays $h(t)$ and τ. The exponential weighting factor $\mathrm{e}^{\alpha(s-t)}$ helps in emphasizing more recent states, which is crucial for systems with memory effects like delays.

Advanced Handling of Delayed States in $V_4(t)$:

$V_4(t)$ includes a double integral structure that considers the interaction between the state derivatives and the state at past delayed times, providing a robust way to account for the system's response over time. The use of matrices Q_{11}, Q_{12}, Q_{22} within this integral allows for a nuanced stability analysis that takes into account the correlation between different states and their derivatives across different delays.

These elements of the Lyapunov functional are specifically designed to address the challenges posed by systems with neutral-type characteristics and time-varying delays. They

ensure that the system's stability can be analyzed comprehensively, considering both immediate and past dynamics. This approach is particularly novel in its integration of complex delay structures into the stability analysis, which is not commonly addressed in traditional Lyapunov functionals used for simpler systems without such intricate delay dynamics.

We believe these explanations highlight the innovative aspects of our Lyapunov functional and its suitability for analyzing the stability of complex systems described by equation (2). Thank you once again for allowing us to clarify these points.

The authors would like to extend our heartfelt gratitude to the reviewer for their thorough and insightful comments, which have been instrumental in enhancing the quality and clarity of our manuscript. We deeply value the time and effort they have dedicated to providing constructive feedback and thoughtful suggestions, which have helped us to refine the content and presentation of our work further.

References

[1] Pin-Lin Liu. Improved results on delay-interval-dependent robust stability criteria for uncertain neutral-type systems with time-varying delays. *Isa Transactions*, 60:53–66, 2016.

[2] Li Wan, Qinghua Zhou, Hongbo Fu, and Qunjiao Zhang. Exponential stability of hopfield neural networks of neutral type with multiple time-varying delays. *AIMS Math*, 6(8):8030–8043, 2021.

[3] Xiaohui Duan, Fangping Tang, and Wenyong Duan. Improved robust stability criteria for uncertain linear neutral-type systems via novel lyapunov-krasovskii functional. *Asian Journal of Control*, 22(2):976–987, 2020.

[4] Wim Michiels and Tomáš Vyhlídal. An eigenvalue based approach for the stabilization of linear time-delay systems of neutral type. *Automatica*, 41(6):991–998, 2005.

[5] Zahra Sadat Aghayan, Alireza Alfi, and JA Tenreiro Machado. Robust stability of uncertain fractional order systems of neutral type with distributed delays and control input saturation. *ISA transactions*, 111:144–155, 2021.

[6] Mouquan Shen, Xianming Wang, Ju H Park, Yang Yi, and Wei-Wei Che. Extended disturbance-observer-based data-driven control of networked nonlinear systems with event-triggered output. *IEEE Transactions on Systems, Man, and Cybernetics: Systems*, 53(5):3129–3140, 2022.

[7] Xiangli Jiang, Guihua Xia, Zhiguang Feng, Zhengyi Jiang, and Jianbin Qiu. Reachable set estimation for markovian jump neutral-type neural networks with time-varying delays. *IEEE Transactions on Cybernetics*, 52(2):1150–1163, 2020.

[8] Changchun Shen, Shouwei Zhou, Hongyong Deng, et al. The no-ellipsoidal bound of reachable sets for neutral markovian jump systems with disturbances. *Journal of Applied Mathematics and Physics*, 8(05):799, 2020.

[9] Xipan Zhang, Changchun Shen, and Dingju Xu. Reachable set estimation for neutral semi-markovian jump systems with time-varying delay. *AIMS Mathematics*, 9(4):8043–8062, 2024.

[10] Mouquan Shen, Hainan Zhang, Sing Kiong Nguang, and Choon Ki Ahn. H∞ output anti-disturbance control of stochastic markov jump systems with multiple disturbances. *IEEE Transactions on Systems, Man, and Cybernetics: Systems*, 51(12):7633–7643, 2020.

[11] Zhiguang Feng, Wei Xing Zheng, and Ligang Wu. Reachable set estimation of t–s fuzzy systems with time-varying delay. *IEEE Transactions on Fuzzy Systems*, 25(4):878–891, 2016.

[12] Zhiqiang Zuo, Yinping Chen, Yijing Wang, Daniel WC Ho, Michael ZQ Chen, and Hongchao Li. A note on reachable set bounding for delayed systems with polytopic uncertainties. *Journal of the Franklin Institute*, 350(7):1827–1835, 2013.

[13] Jack K Hale and Kenneth R Meyer. *A class of functional equations of neutral type*, volume 76. American Mathematical Society Providence, RI, USA, 1967.

[14] Changchun Shen and Shouming Zhong. The ellipsoidal bound of reachable sets for linear neutral systems with disturbances. *Journal of the Franklin Institute*, 348(9):2570–2585, 2011.

[15] Liang Zhang, Xudong Zhao, and Ning Zhao. Real-time reachable set control for neutral singular markov jump systems with mixed delays. *IEEE Transactions on Circuits and Systems II: Express Briefs*, 69(3):1367–1371, 2021.

[16] Zahra Sadat Aghayan, Alireza Alfi, Yashar Mousavi, and Afef Fekih. Stability analysis of a class of variable fractional-order uncertain neutral-type systems with time-varying delay. *Journal of the Franklin Institute*, 360(14):10517–10535, 2023.

[17] Gerson Portilla, Irina V Alexandrova, and Sabine Mondié. Stability tests for neutral-type delay systems: A delay lyapunov matrix and piecewise linear approximation of the functional argument approach. *International Journal of Robust and Nonlinear Control*, 2024.

[18] Leonid Berezansky, Josef Diblík, Zdeněk Svoboda, and Zdeněk Šmarda. Exponential stability criteria for linear neutral systems with applications to neural networks of neutral type. *Journal of the Franklin Institute*, 360(1):301–326, 2023.

[19] Jin-Hoon Kim. Improved ellipsoidal bound of reachable sets for time-delayed linear systems with disturbances. *Automatica*, 44(11):2940–2943, 2008.

[20] Zhiqiang Zuo, Daniel WC Ho, and Yijing Wang. Reachable set bounding for delayed systems with polytopic uncertainties: the maximal lyapunov–krasovskii functional approach. *Automatica*, 46(5):949–952, 2010.

[21] Chunbin Qin, Tianzeng Zhu, Kaijun Jiang, and Yinliang Wu. Integral reinforcement learning-based dynamic event-triggered safety control for multiplayer stackelberg–nash games with time-varying state constraints. *Engineering Applications of Artificial Intelligence*, 133:108317, 2024.

[22] Chunbin Qin, Ziyang Shang, Zhongwei Zhang, Dehua Zhang, and Jishi Zhang. Parallel learning-based security robust tracking control for nonlinear systems with uncertainties: An event-triggered design. *Engineering Applications of Artificial Intelligence*, 133:108077, 2024.

[23] Chunbin Qin, Xiaopeng Qiao, Jinguang Wang, Dehua Zhang, Yandong Hou, and Shaolin Hu. Barrier-critic adaptive robust control of nonzero-sum differential games for uncertain nonlinear systems with state constraints. *IEEE Transactions on Systems, Man, and Cybernetics: Systems*, 2023.
